# Association between statin use and acute pulmonary embolism in intensive care unit patients with sepsis: a retrospective cohort study

**DOI:** 10.3389/fmed.2024.1369967

**Published:** 2024-04-08

**Authors:** Dengcang Yang, Yanyan He, Qianqian Wang, Yi Yu

**Affiliations:** ^1^Anesthesiology Department, The Central Hospital of Shaoyang, Shaoyang, Hunan, China; ^2^Department of Geriatric Medicine, The Second Affiliated Hospital of Shaoyang University, Shaoyang, Hunan, China; ^3^Department of Pulmonary and Critical Care Medicine, The First Affiliated Hospital, Sun Yat-Sen University, Guangzhou, Guangdong, China; ^4^Department of Pulmonary and Critical Care Medicine, Guangxi Hospital Division of The First Hospital, Sun Yat-Sen University, Nanning, Guangxi Zhuang, China; ^5^Department of Critical Care Medicine, The Second Affiliated Hospital of Guangzhou University of Chinese Medicine, Guangzhou, Guangdong, China

**Keywords:** statins, acute pulmonary embolism, ICU, medical information mart for intensive care, cohort study

## Abstract

**Introduction:**

Acute pulmonary embolism (APE) is a life-threatening medical condition that is frequently encountered and associated with significant incidence and mortality rates, posing a substantial threat to patients’ well-being and quality of life. Sepsis is prominent independent risk factor for the development of APE. Despite recent investigations indicating a reduced APE risk through statin therapy, its impact on patients with sepsis and APE remains unresolved.

**Methods:**

The Medical Information Mart for Intensive Care (MIMIC)-IV database was utilized to identify patients diagnosed with sepsis and APE, irrespective of statin treatment status, as part of this study. The primary study aim was to assess the risk of APE, which was analyzed using multivariate logistic regression models.

**Results:**

The study encompassed a total of 16,633 participants, with an average age of 64.8 ± 16.2 years. Multivariate logistic regression revealed that septic patients receiving statin therapy in the intensive care unit (ICU) exhibited a 33% reduction in the risk of developing APE (OR = 0.67, 95% CI: 0.52–0.86, *p* < 0.001). The findings of further analyses, including stratification based on statin usage, dosage, and propensity score matching, consistently reinforced the hypothesis that administering statins to patients with sepsis effectively mitigates their potential APE risk.

**Discussion:**

The results of the study provide compelling evidence in favor of administering statins to septic patients as a prophylactic measure against APE, given that statins may reduce the risk of developing APE, and their anti-APE effect appears to be dose-dependent. Nonetheless, future randomized controlled trials are needed to validate these results.

## Introduction

Acute pulmonary embolism (APE), which is classified as venous thromboembolism (VTE), is a cardiovascular disorder characterized by its high rates of occurrence and mortality, ranking closely behind myocardial infarction and stroke. Despite its notable prevalence and fatality, APE continues to be under-diagnosed, which poses a substantial risk to patients’ overall well-being and quality of life (1, 2).

The increased incidence of APE observed in critically ill patients is attributed to various factors, including complete immobilization, reluctance to administer anticoagulant prophylaxis due to a heightened risk of bleeding, and impaired peripheral circulation in patients receiving vasopressor drugs to sustain central blood pressure, thereby leading to reduced subcutaneous heparin bioavailability (3, 4). Furthermore, sepsis is a notable stand alone risk factor for the development of APE (5–7). The initial phases of sepsis involve a multitude of concurrent pathophysiological mechanisms, encompassing inflammation and activation of coagulation pathways (8). The coagulation cascade is an intricately regulated process, and changes in patients’ coagulation profile during sepsis are indicative of an adverse prognostic outcome (9). Additionally, individuals with sepsis display decreased concentrations of antifactor Xa, due to inflammation, tissue permeability, and pronounced subcutaneous edema, compared to a control group without edema (10, 11). Although long-term use of vitamin K antagonists effectively reduces the risk of VTE in high-risk individuals, it is associated with an increased likelihood of experiencing major hemorrhagic events (12, 13). In light of these factors, critically ill patients, especially those diagnosed with sepsis, are confronted with the pivotal issue of identifying secure alternatives to effectively manage the risk of APE, when conventional anticoagulation therapies and oral anticoagulants are either ineffective or contraindicated.

Statins are widely employed for the prophylaxis and management of atherosclerotic ailments both in primary and secondary settings (14). Current evidence suggests there is a common mechanism that underlies both VTE and atherosclerotic disease (15–17); e.g., cytokines released by inflammatory cells, which have been detected in atherosclerotic plaques, have also been identified in individuals suffering from venous thrombosis (18). Aside from their lipid-lowering properties, statins exhibit a spectrum of vasoprotective actions that could bolster the possible utility of statin therapy in the treatment of VTE (19, 20). Violi et al. published a review paper summarizing the positive impact of statins on the vascular wall, inflammation, and thrombotic factors, which collectively demonstrated a vasoprotective effect (21).

Given the existing evidence, our hypothesis was that statins have a role in preventing APE in high-risk patients admitted to the intensive care unit (ICU). Therefore, we conducted a retrospective study of a cohort of 16,633 critically ill patients. The data used for this study were obtained from the Medical Information Mart for Intensive Care (MIMIC-IV) dataset covering the period from 2001 to 2019. Our objective was to investigate the association between the use of statins and the risk of APE in ICU sepsis patients.

## Materials and methods

This study used data from patients diagnosed with sepsis and APE (regardless of their prior use of statins) that were retrieved from the MIMIC-IV (version 2.2) database, which is a comprehensive and longitudinal collection of patients’ information from a single healthcare center. The database encompasses data recorded between 2008 and 2019 (22); prior authorization to use the database was obtained from Yi Yu, who is one of the authors (certificate ID number 6477678). This study complies with the Guidelines for Strengthening the Reporting of Observational Studies in Epidemiology (STROBE) (23).

### Study sample and data extraction

The study enrolled individuals with a confirmed diagnosis of APE with sepsis based on their discharge diagnosis. The diagnosis of sepsis is based on the Sepsis 3.0 criteria. Sepsis was defined as life-threatening organ dysfunction caused by a dysregulated host response to infection. For clinical operationalization, organ dysfunction was represented by an increase in the Sequential (Sepsis-related) Organ Failure Assessment (SOFA) score of 2 points or more (24). The inclusion criteria were: (1) APE had to be listed among the top five discharge diagnoses and had to be explicitly mentioned in the discharge diagnosis; and (2) the patients had to be 18 years of age or older. In cases where patients had multiple ICU admissions, only data from the initial admission were used. A comprehensive set of patients’ data was collected, including demographic details, vital signs, underlying conditions, laboratory results, clinical severity scores, and additional admission information. The diagnosis of APE was determined using the International Classification of Diseases, 9th and 10th editions.

### Statin use

The presence of statin medications in the “prescriptions” data from the MIMIC-IV database was used to assess the administration of statins. The statins included in the analyses were atorvastatin, simvastatin, rosuvastatin, lovastatin, pravastatin, and fluvastatin. The average daily dose was calculated to determine the dosage of statins. The classification of statin dosages was based on the potency of each statin, as indicated on a standard conversion chart (25).

### Covariates

The database contained variables previously reported to be cardiovascular risk factors and potential triggers for APE, as well as other variables (26–29). Personal demographic variables included age, sex, race, and body mass index (BMI). Health related variables included: respiratory rate, body temperature, the Saturation of Peripheral Oxygen ratio (SPO2), Sequential Organ Failure Assessment (SOFA) score, White Blood Cell (WBC) count, hemoglobin level, hematocrit, platelet count, and glucose level, and preexisting medical conditions (e.g., cardiovascular disease, kidney disease, rheumatic disease, liver disease, cancer, neurological disease, and chronic pulmonary disease).

### Outcome

The outcome variable was the probability of developing APE.

### Statistical analysis

The study initially analyzed the baseline characteristics of the total sample and compared the characteristics of the two cohorts (Statin use and No statin use). Categorical data are summarized as frequency counts and percentages, whereas continuous data are presented as mean ± standard deviation or median (interquartile range), where appropriate. Analysis of variance or rank sum tests were performed to analyze differences in cohort outcomes for continuous variables. The Chi-square or Fisher’s exact tests were performed to analyze group (i.e., cohort) differences in outcomes for categorical variables.

We used a median replacement strategy to impute missing data on vital signs and laboratory parameters, as these variables contained missing data in 5% of the sample. Since the percentage of missing data for height and weight was low (ranging from 0.3–4%), no imputation was performed. We initially tested five multivariate logistic regression models to analyze the unique association between statins and APE, adjusting for different covariates. We performed some different statistical models to verify the results’ stability. In the final model, we adjusted the factors basing the following three rules (1 or 2 or 3). (1) We adjusted for variables, if it was added to this model, the matched odds ratio would change at least 10%. (2) For univariate analysis, we adjusted for variables, of which the *p* values were <0.1. (3) For multivariable analysis, variables were chosen on the basis of previous findings and clinical constraints. Supplementary analyses were conducted to examine subgroup and interaction analyses, controlling for relevant covariates. Propensity score matching (PSM) was conducted to improve the rigor of the study, using a 1:1 nearest neighbor matching algorithm with a caliper width of 0.1. Multivariate logistic regression models with robust variance estimators were employed to estimate the odds ratio (OR) for APE.

The statistical analyses were performed with STATA software (version 17.0), R packages (The R Foundation),^1^ and Free Statistics software version 1.8 (30). Statistical significance was set to *p* < 0.05 (two-tailed).

## Results

### Participants

**Figure 1:**
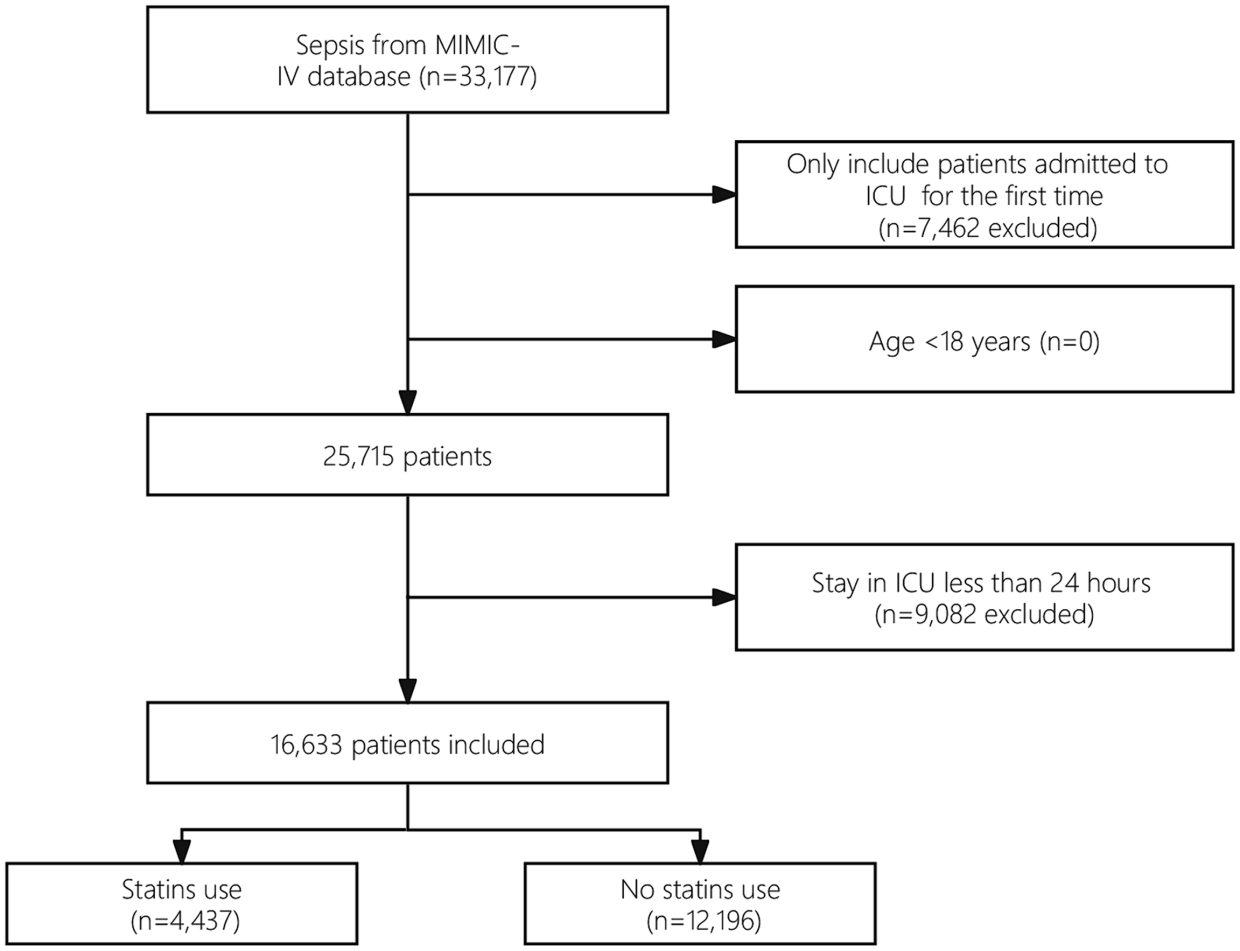
Study flowchart.

Among the eligible patients, a total of 33,177 individuals met the sepsis criteria. After excluding cases of repeated ICU admissions and patients with an ICU stay of less than 24 h, the final cohort included 16,633 patients. Figure 1 presents a flowchart that illustrates the process of selecting study participants.

### Baseline characteristics

A total of 16,633 patients (57.3% male, mean age = 64.8 ± 16.2 years) were selected for inclusion in the study. The baseline characteristics of the study sample are presented in Table 1. Comparisons between the two groups indicated that the non-statin group was younger and had (a) a higher proportion of females, (b) higher SOFA scores, (c) a lower Charlson comorbidity index, (d) higher liver disorders rates and (e) significantly higher rates of APE, deep vein thrombosis (DVT), 30- and 90-day mortality. Longer ICU stays also were observed in the non-statin group. In the statins group, there were no significant liver or muscle-related side effects.

**Table 1:** Baseline characteristics of the participants.

Variables	Total (*N* = 16,633)	No statin use (*n* = 12,196)	Statin use (*n* = 4,437)	*p* value
Age, years	64.8 ± 16.2	62.7 ± 17.0	70.6 ± 12.1	<0.001
Sex, Male, *n* (%)	9,539 (57.3)	6,864 (56.3)	2,675 (60.3)	<0.001
BMI, kg/m^2^	29.1 ± 7.4	29.1 ± 7.6	29.4 ± 6.9	0.027
Race, *n* (%)				<0.001
White	11,116 (66.8)	8,098 (66.4)	3,018 (68)	
Black	1,503 (9.0)	1,264 (10.4)	239 (5.4)	
Others	4,014 (24.1)	2,834 (23.2)	1,180 (26.6)	
Hematocrit (%)	32.0 ± 6.1	31.8 ± 6.2	32.3 ± 5.8	<0.001
Hb (g/L)	10.5 ± 2.1	10.4 ± 2.1	10.7 ± 2.0	<0.001
PLT (×10^9^)	207.5 ± 114.5	208.9 ± 120.4	203.7 ± 96.6	0.009
WBC (×10^9^)	13.5 ± 9.5	13.6 ± 10.2	13.5 ± 7.1	0.642
Respiration rate (bpm)	20.0 ± 4.1	20.1 ± 4.2	19.4 ± 3.7	<0.001
Temperature (°C)	36.9 ± 0.7	36.9 ± 0.7	36.8 ± 0.7	<0.001
SPO_2_ (%)	97.1 ± 2.2	97.1 ± 2.2	97.1 ± 2.0	0.238
Glucose (mmol/L)	144.7 ± 46.0	143.8 ± 46.6	146.9 ± 43.9	<0.001
Charlson comorbidity index	6.6 ± 3.2	6.6 ± 3.4	6.8 ± 2.6	<0.001
SOFA score	6.2 ± 3.4	6.4 ± 3.6	5.9 ± 3.0	<0.001
Myocardial infarct, *n* (%)	3,422 (20.6)	1909 (15.7)	1,513 (34.1)	<0.001
Congestive heart failure, *n* (%)	6,060 (36.4)	4,124 (33.8)	1936 (43.6)	<0.001
Peripheral vascular disease, *n* (%)	2,576 (15.5)	1731 (14.2)	845 (19)	<0.001
Cerebrovascular disease, *n* (%)	3,230 (19.4)	2,196 (18)	1,034 (23.3)	<0.001
Chronic pulmonary disease, *n* (%)	5,149 (31.0)	3,743 (30.7)	1,406 (31.7)	0.218
Rheumatic disease, *n* (%)	664 (4.0)	512 (4.2)	152 (3.4)	0.024
Malignant cancer, *n* (%)	2,770 (16.7)	2,283 (18.7)	487 (11)	<0.001
Severe liver disease, *n* (%)	1,437 (8.6)	1,344 (11)	93 (2.1)	<0.001
hypertension, *n* (%)	4,551 (27.4)	3,014 (24.7)	1,537 (34.6)	<0.001
Diabetes, *n* (%)				<0.001
None	11,103 (66.8)	8,411 (69)	2,692 (60.7)	
Without complications	3,307 (19.9)	2,148 (17.6)	1,159 (26.1)	
With complications	2,223 (13.4)	1,637 (13.4)	586 (13.2)	
ALT (U/L)^*^	50.0 (27.0, 125.0)	52.0 (29.0, 143.0)	39.0 (22.0, 82.0)	<0.001
AST (U/L)^*^	67.0 (35.0, 173.0)	69.0 (38.0, 198.0)	53.0 (29.0, 114.0)	<0.001
CK (U/L)^*^	198.0 (114.0, 367.0)	198.0 (110.0, 374.0)	198.0 (127.0, 350.0)	0.023
ICU stay, days	4.5 (2.9 ± 8.5)	4.6 (2.9 ± 8.7)	4.4 (2.9 ± 8.1)	0.01
30-Day mortality, *n* (%)	2,901 (17.4)	2,361 (19.4)	540 (12.2)	<0.001
90-Day mortality, *n* (%)	3,153 (19.0)	2,578 (21.1)	575 (13)	<0.001
DVT, *n* (%)	299 (1.8)	264 (2.2)	35 (0.8)	<0.001
Acute pulmonary embolism, *n* (%)	493 (3.0)	396 (3.2)	97 (2.2)	<0.001

Mean ± standard deviation, median (interquartile range), or number (percentage) is reported for each variable, as appropriate. BMI, body mass index; HB, hemoglobin; Plt, platelets; WBC, white blood cells; SPO_2_, pulse oxygen saturation; SOFA, Sequential Organ Failure Assessment; ALT, alanine transaminase; AST, aspartate transaminase; CK, creatine kinase; ICU, intensive care unit; DVT, deep vein thrombosis. ^*^The maximum levels during the patient’s stay in the ICU.

### Relationship between statin use and APE and DVT

The univariate analyses found that the use of statins significantly reduced the rates of APE compared to no statin use (OR = 0.67, 95% confidence interval = 0.53–0.83, *p* < 0.001; Table 2).

**Table 2:** Statin use for APE.

	OR of statin use	95% CI	*p* value
Model 1	0.67	0.53–0.83	<0.001
Model 2	0.75	0.6–0.95	<0.001
Model 3	0.76	0.6–0.96	<0.001
Model 4	0.65	0.51–0.82	<0.001
Model 5	0.67	0.52–0.86	<0.001
PSM	0.68	0.52–0.9	0.007

OR, odds ratio; CI, confidence interval; PSM, propensity score matching. Model 1: not adjusted. Model 2: age, sex, BMI. Model 3: Model 2, race, glucose, WBC, PLT, hematocrit. Model 4: Model 3, SOFA score, deep venous thrombosis, ICU stay, aspirin use, oral anticoagulant. Model 5: Model 4, DM, hypertension, malignant cancer, peripheral vascular disease, chronic pulmonary disease, severe liver disease, renal disease, cerebrovascular disease, congestive heart failure.

The multivariate logistic regression analyses (Table 2) found the ORs for the benefit of using statins remained consistently significant across all five models (ORs ranged from 0.65 to 0.76, *p* < 0.05 for all models). Model 5, which controlled for all the covariates, found the use of statins had a significant 33% reduction in APE risk (OR = 0.67, *p* < 0.001), and these results were robust.

The multivariate logistic regression analyses for DVT of using statins remained consistently significant across all five models (ORs ranged from 0.36 to 0.53, *p* < 0.05 for all models). Model 5, which controlled for all the covariates, found the use of statins had a significant 47% reduction in DVT risk (OR = 0.53, *p* < 0.001; Supplementary Table S2).

### Subgroup analysis and sensitivity analysis

**Figure 2:**
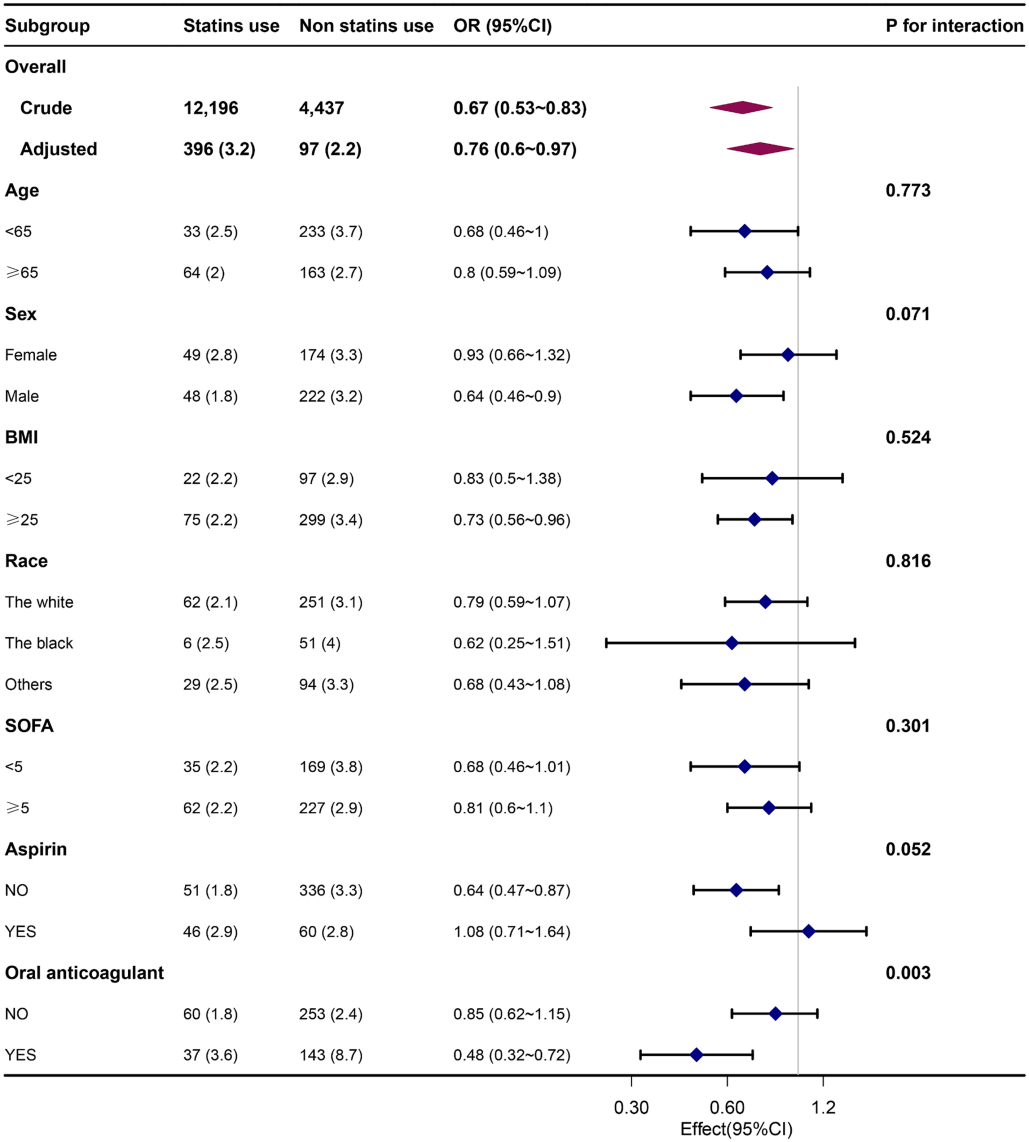
Association between statin use and APE by patients’ characteristics at baseline. Each stratification was adjusted for all the covariates except the stratification variable itself. OR, odds ratio; BMI, body mass index; SOFA, Sequential Organ Failure Assessment.

The results remained consistent across the logistic regression models. After PSM was conducted on both groups, the sample consisted of 4,437 well-matched pairs, and there were no significant differences in key variables between the two matched groups (Supplementary Table S1). Among the 4,437 pairs in the propensity-matched pool, the risk of APE was significantly lower in patients who were prescribed statins [97 (2.2%) versus 141 (3.2%), *p* = 0.004]. The multivariate logistic regression model that was adjusted for all the covariates yielded an OR = 0.68 (*p* < 0.007) for APE (Table 2). Furthermore, when analyzing the net effect of the dosage of statins, both the standard dose of statin (OR = 0.72) and the high dose of statin (OR = 0.65) were associated with a reduced risk of APE (Table 3). Similarly, when analyzing the classification of statins, atorvastatin (OR = 0.62) had a protective effect, while simvastatin (OR = 0.87) and other statins (OR = 0.54) did not show a significant association with APE (Table 4). Subgroup analysis further supported the robustness and reliability of the observed statin-APE relationship. The protective effects of statins in these subgroup analyses were more pronounced in patients who also used oral anticoagulants than in patients who used non-oral anticoagulants. No other significant interaction was observed in the subgroup analyses (*p* for interaction >0.05) (Figure 2).

**Table 3:** Dosage of statin use for APE.

		Model 1		Model 2		PSM		
Variable	N	OR (95%CI)	*p* value	OR (95%CI)	*p* value	N	OR (95%CI)	*p* value
No use	12,196	1 (Ref)		1 (Ref)		4,435	1 (Ref)	
Standard dose	1,866	0.69 (0.5–0.95)	0.022	0.7 (0.5–0.98)	0.038	1,865	0.72 (0.5–1.03)	0.076
High dose	2,571	0.65 (0.49–0.87)	0.003	0.65 (0.48–0.88)	0.005	2,570	0.65 (0.46–0.91)	0.012
Trend test			<0.001		0.002			0.008

APE, acute pulmonary embolism; OR, odds ratio; Ref, Reference; PSM, propensity score matching; CI, confidence interval. Model 1: not adjusted. Model 2: adjusted for age, sex, BMI, race, glucose, WBC, PLT, hematocrit, SOFA score, deep venous thrombosis, ICU stay, aspirin use, oral anticoagulant, DM, hypertension, malignant cancer, peripheral vascular disease, chronic pulmonary disease, severe liver disease, renal disease, cerebrovascular disease and congestive heart failure.

**Table 4:** Statin classifications for APE.

		Model 1		Model 2		PSM		
Variable	N	OR (95%CI)	*p* value	OR (95%CI)	*p* value	N	OR (95%CI)	*p* value
None	12,196	1 (Ref)		1 (Ref)		4,435	1 (Ref)	
Atorvastatin	2,584	0.62 (0.47 ~ 0.83)	0.001	0.62 (0.45 ~ 0.84)	0.002	2,583	0.62 (0.44–0.87)	0.006
Simvastatin	1,220	0.85 (0.6 ~ 1.22)	0.385	0.85 (0.59 ~ 1.23)	0.95	1,220	0.87 (0.59–1.29)	0.488
Others	633	0.48 (0.25 ~ 0.9)	0.022	0.52 (0.27 ~ 1)	0.048	632	0.54 (0.28–1.03)	0.062

APE, acute pulmonary embolism; OR, odds ratio; Ref, Reference; PSM, propensity score matching; CI, confidence interval. Model 1: not adjusted. Model 2: adjusted for age, sex, BMI, race, glucose, WBC, PLT, hematocrit, SOFA score, deep venous thrombosis, ICU stay, aspirin use, oral anticoagulant, DM, hypertension, malignant cancer, peripheral vascular disease, chronic pulmonary disease, severe liver disease, renal disease, cerebrovascular disease and congestive heart failure.

## Discussion

### The main result

This current study builds upon previous promising findings about the use of statins for patients with sepsis. By utilizing a large-scale database, this study provides robust evidence supporting the favorable effects of statins in reducing the occurrence of APE in sepsis patients. The results of this study validate that administering statins is significantly associated with a substantial reduction in the likelihood of APE in sepsis patients. In addition, the subcategorization of statin usage, analysis of statin dosage, and PSM further strengthen the validity of these findings, by consistently demonstrating the protective effect of statins in reducing the risk of APE in patients with sepsis.

### Effects of statins use on the APE risk of sepsis patients

Extensive research has examined the impact of statins on APE (31–34), and the findings consistently demonstrate that statins effectively mitigate the occurrence of APE in populations at high risk, while also reducing mortality rates associated with APE. Consistent findings were observed in our study, revealing a significant association between the use of statins and a decreased risk of APE in sepsis patients (OR = 0.67). However, it is crucial to note that some studies have not replicated our findings. One study of the administration of statins within the initial year following a successful kidney transplant reported that statins did not reduce the likelihood of PE (35), and a study by Huerta et al. found no substantial protective effect of statins on APE or deep vein thrombosis in the context of current or past statin use (36). While a randomized clinical trial reported that the use of rosuvastatin substantially reduced the occurrence of symptomatic venous thromboembolism, its use did not result in a decline in the frequency of APE (37). However, that study had some limitations, including its sole focus on individuals without existing health issues, and its limited duration of observation. Furthermore, it did not examine the link between statin dosage and the probability of VTE. Another study demonstrated that the efficacy of statin therapy in preventing thrombus formation in cancer patients remains unclear (38). But, it is worth noting that the study only included participants with progressive tumor growth. Additionally, its small sample size and short duration of monitoring were study limitations. Therefore, additional clinical studies are needed to assess the efficacy of statins in the prevention and treatment of APE.

The precise mechanism through which statin use is associated with a reduced risk of APE in patients with sepsis remains unclear. However, apart from their lipid-lowering effect, statins also possess anti-inflammatory properties, leading to decreased levels of inflammatory markers in the blood and improved endothelial function. Moreover, statins exert antithrombotic effects and can regulate coagulation cascades through various mechanisms that are independent of alterations in cholesterol levels (39, 40). The protective effect of simvastatin on APE-induced pulmonary arterial pressure, hypoxemia, and inflammatory changes may be attributed to its modulation of the signaling pathway involving silent information regulator 2 (SIRT2) and nuclear factor-kappa B (NF-κB). Additionally, pretreatment with atorvastatin has been found to improve APE-induced pulmonary hypertension and increase 24-h survival rates by reducing the elevation of lung-activated matrix metalloprotein-9 following APE (41). The promising protective effects of simvastatin in patients with APE are linked to its modulation of the SIRT2/NF-κB signaling pathway. This is supported by its capacity to alleviate APE-induced pulmonary artery pressure, hypoxemia, and inflammatory changes, highlighting its potential therapeutic benefits (42).

### Strengths and limitations

Our study possesses several noteworthy strengths. First, it is worth noting, for example, though the effects of statin administration on APE have been extensively explored, there is a lack of conclusive evidence, specifically, patients with sepsis. Our findings shed light on the substantial reduction in APE risk associated with statin usage in sepsis patients. Second, the rationale for selecting statins lies in their widespread acceptance and ease of use within the medical community. Prior research has demonstrated the broad applicability of statins in the management and prevention of diverse conditions, including tumors, cardiovascular disease, and cerebrovascular disease (43–46). Third, we conducted several sensitivity analyses to ensure the robustness of our results.

These analyses are important for at least four reasons: (1) logistic regression analyses were adjusted using multiple models to control for potential confounding variables, and the stability of the results was confirmed using thorough model adjustments; (2) the analysis of the net effects of standard and high doses of statin, produced reliable findings, with the trend test indicating a more pronounced effect for high-dose administration; (3) the categorization of statin usage into non-use, atorvastatin, simvastatin, and others, revealed the protective effects of various statins against APE; and (4) the employment of PMS analysis yielded results consistent with those of the initial analyses.

We acknowledge several limitations of our study in line with previous observational studies. First, large amounts of missing data prevented us from conducting statistical analyses on lipid levels; therefore the optimal lipid value for APE in sepsis remains unknown. Second, the retrospective nature of our study and unmeasured confounders may have affected our findings. Furthermore, our analysis of serum markers of inflammation was limited to WBC. Interleukin-6, C-reactive protein, and procalcitonin were not measured due to a large proportion of missing values. A significant amount of data is also missing for risk stratification factors related to APE (echocardiography, electrocardiogram, CT - scan, BNP, and TNT). Third, our study may not be generalizable as it was conducted in a single institution in the United States. However, our substantial sample size and representative cohort lend support to our findings. Future prospective studies across multiple centers should help validate our findings. Fourth, we were not able to control for several potential confounding variables, such as smoking history, drinking history, hormone use, and other medical histories, which may have influenced the risk of APE in patients with sepsis. Additionally, the retrospective nature of our study prevents us from providing detailed information on the impact of statins on individual patients’ lipid levels, such as dosage and duration of use.

## Conclusion

The current evidence on the use of statins in sepsis patients shows that statins may reduce the incidence of APE and that they may also have a dose-related anti-APE effect. Nonetheless, there is a need for future randomized controlled trials to validate the claims made in this manuscript.

## Data availability statement

The raw data supporting the conclusions of this article will be made available by the authors, without undue reservation.

## Ethics statement

The studies involving human participants were reviewed and approved by the Massachusetts Institute of Technology and Beth Israel Deaconess Medical Center. Written informed consent to participate in this study was provided by the participants’ legal guardian/next of kin. The studies were conducted in accordance with the local legislation and institutional requirements. The participants provided their written informed consent to participate in this study.

## Author contributions

DY: Formal analysis, Investigation, Software, Writing – original draft. YH: Conceptualization, Data curation, Methodology, Supervision, Writing – original draft. QW: Conceptualization, Formal analysis, Investigation, Project administration, Validation, Visualization, Writing – original draft. YY: Writing – review & editing.

## Footnotes

^1^http://www.R-project.org
