# Supplementary material for: Association between statin use and acute pulmonary embolism in intensive care unit patients with sepsis: a retrospective cohort study
Source: Front Med (Lausanne). 2024 Apr 8;11:1369967. doi: 10.3389/fmed.2024.1369967 (PMC11033833; doi:10.3389/fmed.2024.1369967)
Supplement: Supplementary file 1 [file Table_1.docx]

**Table 1S.** Characteristics of participants at baseline after PSM

| Variables | Total (n = 8,870) | No statin use  (n = 4,437) | Statin use  (n = 4,437) | P value |
| --- | --- | --- | --- | --- |
| Age, years | 70.9 ± 13.0 | 71.1 ± 13.9 | 70.6 ± 12.1 | 0.062 |
| Sex, Male, n (%) | 5343 (60.2) | 2669 (60.2) | 2674 (60.3) | 0.914 |
| BMI, kg/m^2^ | 29.3 ± 7.2 | 29.3 ± 7.6 | 29.4 ± 6.9 | 0.633 |
| Race, n (%) |  |  |  | < 0.001 |
| White | 6102 (68.8) | 3085 (69.6) | 3017 (68) |  |
| Black | 645 ( 7.3) | 406 (9.2) | 239 (5.4) |  |
| Others | 2123 (23.9) | 944 (21.3) | 1179 (26.6) |  |
| Hematocrit (%) | 32.4 ± 5.9 | 32.4 ± 6.0 | 32.3 ± 5.8 | 0.619 |
| Hb (g/L) | 10.6 ± 2.0 | 10.6 ± 2.1 | 10.7 ± 2.0 | 0.065 |
| PLT (×10^9^ ) | 205.6 ± 101.8 | 207.4 ± 106.7 | 203.7 ± 96.6 | 0.09 |
| WBC (×10^9^ ) | 13.5 ± 9.3 | 13.6 ± 11.0 | 13.5 ± 7.1 | 0.556 |
| Respiration rate (bpm) | 19.8 ± 3.9 | 20.1 ± 4.0 | 19.4 ± 3.7 | < 0.001 |
| Temperature(°C) | 36.9 ± 0.7 | 36.9 ± 0.6 | 36.8 ± 0.7 | 0.004 |
| SPO_2_ (%) | 97.1 ± 2.1 | 97.0 ± 2.1 | 97.1 ± 2.0 | 0.129 |
| Glucose (mmol/L) | 146.9 ± 45.1 | 146.9 ± 46.3 | 146.9 ± 43.9 | 0.978 |
| Charlson comorbidity index | 6.8 ± 2.7 | 6.9 ± 2.8 | 6.8 ± 2.6 | 0.188 |
| SOFA score | 5.9 ± 3.0 | 5.8 ± 3.1 | 5.9 ± 3.0 | 0.195 |
| Myocardial infarct, n (%) | 2250 (25.4) | 737 (16.6) | 1513 (34.1) | < 0.001 |
| Congestive heart failure, n (%) | 3665 (41.3) | 1730 (39) | 1935 (43.6) | < 0.001 |
| Peripheral vascular disease, n (%) | 1515 (17.1) | 671 (15.1) | 844 (19) | < 0.001 |
| Cerebrovascular disease, n (%) | 1926 (21.7) | 892 (20.1) | 1034 (23.3) | < 0.001 |
| Chronic pulmonary disease, n (%) | 2779 (31.3) | 1373 (31) | 1406 (31.7) | 0.45 |
| Rheumatic disease, n (%) | 330(3.7) | 178 (4) | 152 (3.4) | 0.145 |
| Malignant cancer, n (%) | 1192 (13.4) | 705 (15.9) | 487 (11) | < 0.001 |
| Severe liver disease, n (%) | 344 ( 3.9) | 251 (5.7) | 93 (2.1) | < 0.001 |
| hypertension, n (%) | 2896 (32.6) | 1361 (30.7) | 1535 (34.6) | < 0.001 |
| Diabetes, n (%) |  |  |  | 0.54 |
| None | 5411 (61.0) | 2719 (61.3) | 2692 (60.7) |  |
| Without complications | 2270 (25.6) | 1113 (25.1) | 1157 (26.1) |  |
| With complications | 1189 (13.4) | 603 (13.6) | 586 (13.2) |  |
| ALT (U/L)^*^ | 44.0 (24.0, 94.0) | 49.0 (25.0, 105.0) | 39.0 (22.0, 82.0) | < 0.001 |
| AST (U/L)^*^ | 59.0 (31.0, 125.0) | 66.0 (34.0, 138.0) | 53.0 (29.0, 114.0) | < 0.001 |
| CK (U/L)^*^ | 198.0 (115.0, 334.0) | 198.0 (106.0, 324.0) | 198.0 (127.0, 350.5) | < 0.001 |
| ICU stay, days | 4.4 (2.9, 8.2) | 4.4 (2.9, 8.3) | 4.4 (2.9, 8.1) | 0.698 |
| 30-day mortality, n (%) | 1377 (15.5) | 837 (18.9) | 540 (12.2) | < 0.001 |
| 90-day mortality, n (%) | 1480 (16.7) | 905 (20.4) | 575 (13) | < 0.001 |
| DVT, n (%) | 114 ( 1.3) | 79 (1.8) | 35 (0.8) | < 0.001 |
| Acute Pulmonary embolism, n (%) | 238 ( 2.7) | 141 (3.2) | 97 (2.2) | 0.004 |
| High-risk group for Acute Pulmonary embolism, n (%) | 5547 (62.5) | 2607 (58.8) | 2940 (66.3) | < 0.001 |

Mean ± standard deviation, median (interquartile range), or number (percentage) is reported for each variable, as appropriate.

PSM, Propensity Score Matching; BMI, body mass index; HB, hemoglobin; Plt, platelets; WBC, white blood cells; SPO_2_, pulse oxygen saturation; SOFA, Sequential Organ Failure Assessment; ALT, Alanine Transaminase; AST, Aspartate Transaminase; CK, Creatine Kinase; ICU, Intensive Care Unit; DVT, Deep Vein Thrombosis; ^*^, The maximum levels during the patient's stay in the ICU.
