# Supplementary material for: Association between statin use and acute pulmonary embolism in intensive care unit patients with sepsis: a retrospective cohort study
Source: Front Med (Lausanne). 2024 Apr 8;11:1369967. doi: 10.3389/fmed.2024.1369967 (PMC11033833; doi:10.3389/fmed.2024.1369967)
Supplement: Supplementary file 2 [file Table_2.docx]

**Table 2S.** Statin use for DVT

|  | OR of statin use | 95% CI | P value |
| --- | --- | --- | --- |
| Model 1 | 0.36 | 0.25-0.51) | < 0.001 |
| Model 2 | 0.41 | 0.28-0.58 | < 0.001 |
| Model 3 | 0.42 | 0.29-0.61 | < 0.001 |
| Model 4 | 0.41 | 0.28-0.59 | < 0.001 |
| Model 5 | 0.53 | 0.36-0.77) | 0.001 |
| PSM | 0.6 | 0.4-0.92 | 0.02 |

DVT, deep vein thrombosis; OR, odds ratio; CI, Confidence interval; PSM, Propensity Score Matching.

**Model 1:** Not adjusted.

**Model 2:** Age, sex, BMI.

**Model 3**: Model 2, race, glucose, WBC, PLT, hematocrit.

**Model 4**: Model 3, SOFA score, ICU stay, aspirin use, oral anticoagulant.

**Model 5**: Model 4, DM, hypertension, malignant cancer, peripheral vascular disease, chronic pulmonary disease, severe liver disease, renal disease, cerebrovascular disease, congestive heart failure.
